# Real-world data analysis of patients with cancer of unknown primary

**DOI:** 10.1038/s41598-021-02543-1

**Published:** 2021-11-29

**Authors:** Sora Kang, Jae Ho Jeong, Shinkyo Yoon, Changhoon Yoo, Kyu-pyo Kim, Hyungwoo Cho, Baek-Yeol Ryoo, Jinhong Jung, Jeong Eun Kim

**Affiliations:** 1 Department of Medical Oncology, Asan Medical Center, University of Ulsan College of Medicine, Seoul, South Korea; 2 Department of Radiation Oncology, Asan Medical Center, University of Ulsan College of Medicine, Seoul, South Korea

**Keywords:** Cancer, Oncology

## Abstract

Cancer of unknown primary (CUP) is a heterogeneous malignancy in which the primary site of the tumor cannot be identified through standard work-up. The survival outcome of CUP is generally poor, and there is no consensus for treatment. Here, we comprehensively analyzed the real-world data of 218 patients with CUP (median age, 62 years [range, 19–91]; male, 62.3%). Next-generation sequencing was conducted in 22 (10%) patients, one of whom showed level 1 genetic alteration. Most (60.3%) patients were treated with empirical cytotoxic chemotherapy, and two patients received targeted therapy based on the NGS results. The median OS was 8.3 months (95% confidence interval [CI] 6.2–11.4), and the median progression-free survival of patients treated with chemotherapy was 4.4 months (95% CI 3.4–5.3). In multivariate Cox regression analysis, Eastern Cooperative Oncology Group performance status (ECOG PS) of 0 or 1 and localized disease were significantly associated with favorable survival outcomes. Collectively, we found that CUP patients had a poor prognosis after standard treatment, and those with localized disease who received local treatment and those with better PS treated with multiple lines of chemotherapy had better survival outcomes. Targeted therapies based on NGS results are expected to improve survival outcomes.

## Introduction

Cancer of unknown primary (CUP) is a heterogeneous malignant condition for which the primary site of origin has not been identified by general diagnostic evaluation^1^. CUP historically accounted for 3–5% of all malignancies, but this number has recently decreased to 1–2% owing to advances in diagnostic methods including molecular profiling^2^. Most patients with CUP have been included in unfavorable subsets and showed poor prognosis, with a median overall survival (OS) of 6 months^1^. Because of the heterogeneity of CUP, there has been no consensus on the standard treatment, and empirical cytotoxic chemotherapy and palliative radiotherapy (RT) based on suspected primary sites have been generally used for patients with CUP^3^.

Recently, gene expression profiling and next-generation sequencing (NGS) have gained attention as tools for identifying the primary site and studying the molecular features of CUP^4–7^. Moreover, the number of studies on site-specific therapy and targeted therapy based on gene expression profiling and NGS results in CUP patients has been increasing^8–11^. Some prospective studies have demonstrated better survival outcomes after tailored therapy than after empirical cytotoxic chemotherapy^8,9^, but randomized trials did not report such differences^10,11^. This suggests that there is some discrepancy between studies in terms of the efficacy of the novel targeted therapies for CUP^12^, which is likely due to the incomplete understanding of the characteristics of CUP.

Despite recent advances in diagnostic techniques and treatment strategies, the characteristics of CUP and treatment patterns in real-world settings are not well-known. The clinical utility of tailored therapy is also yet to be established as there are few studies on how novel targeted therapies are being applied in clinical practice. For this reason, we conducted a retrospective study on patients who were diagnosed with CUP and underwent treatment at Asan Medical Center, a 2700-bed tertiary center in Seoul, South Korea, to examine the clinical and molecular characteristics, treatment patterns, survival outcomes, and efficacy of NGS and targeted therapy in patients with CUP in a real-world setting.

## Results

### Patient characteristics

Between January 2009 and December 2019, a total of 218 patients with CUP were registered in the Asan Medical Center cancer registry. During diagnostic work-up, all patients underwent medical history taking, physical examination, baseline blood and biochemistry analyses, imaging studies including chest and abdomen-pelvis computed tomography (CT), and biopsy. Additionally, 94% (n = 206) of patients underwent positron emission tomography (PET)/CT as a part of the diagnostic work-up. Table 1 shows the patient characteristics. The median age was 62 years (range 19–91) and males accounted for 62.3%; 142 (65.1%) patients had an initial Eastern Cooperative Oncology Group Performance Status (ECOG PS) of 0 or 1, and the rest had an initial ECOG PS of 2 or higher. Approximately 85% of patients had disseminated disease, and the median number of metastatic sites was 2 (range, 1–8). In patients with localized disease, the most common metastatic sites were lymph nodes (n = 17, 51.5%) followed by intra-abdominal and pelvic regions (n = 5, 15.2%). In those with disseminated disease, the most common metastatic sites were the bones (46.4%), liver (40%), lung (27%), peritoneum (18.9%), and pleural effusion (10.2%). Approximately 80% (n = 148) of patients showed lymph node metastases. According to the classification of histologic subtypes, carcinoma not otherwise specified (NOS) including poorly differentiated adenocarcinoma accounted for more than half of the patients (n = 122, 55.9%). Squamous cell carcinoma and neuroendocrine tumor accounted for 16% and 13% of the cases, respectively. For patients with neuroendocrine tumors, immunohistochemical staining and imaging studies were not suggestive of pancreatic or gastrointestinal origin.

**Table 1:** Baseline characteristics of the study patients.

Characteristics		Total (n = 218)
**Sex**		
Male		136 (62.4)
Female		82 (37.4)
**Age**		
≥ 60		93 (42.5)
**< **60		125 (57.3)
**ECOG performance status**		
0, 1		142 (65.1)
≥ 2		76 (34.7)
NGS		22 (10.1)
**Disease extent**		
Localized disease		33 (15.1)
Lymph nodes^a^		17 (51.5)
Intra-abdominal, pelvis mass		5 (15.2)
Bone		2 (6.1)
Liver		2 (6.1)
Mediastinal mass		2 (6.1)
Peritoneum carcinomatosis alone		2 (6.1)
Extremity		1 (3.0)
Head and neck lesion		1 (3.0)
Intestine		1 (3.0)
Disseminated disease		185 (84.9)
Lymph nodes		148 (80)
Bone		86 (46.4)
Liver		74 (40)
Intra-abdominal organ except liver^b^		50 (27)
Lung		50 (27)
Peritoneum		35 (18.9)
Pleural effusion		19 (10.2)
Adrenal gland		15 (8.1)
Brain		7 (3.7)
Head and neck lesion		7 (3.7)
Muscle		5 (2.7)
Skin		4 (2.1)

Values are n (%).

*ECOG* Eastern Cooperative Oncology group, *NGS* next-generation sequencing.

^a^Includes cervical (10), Inguinal (5), hepatoduodenal (1) and axillary (1) lymph nodes.

^b^Includes pelvic mass (8), pancreas (7), Gallbladder (7), Ovary (6), intestine (6), stomach (4), intra-abdominal mass (3), spleen(3), kidney and ureter (3), retroperitoneal mass (2), and bladder(1).

### Molecular characteristics of patients with CUP

**Figure 1:**
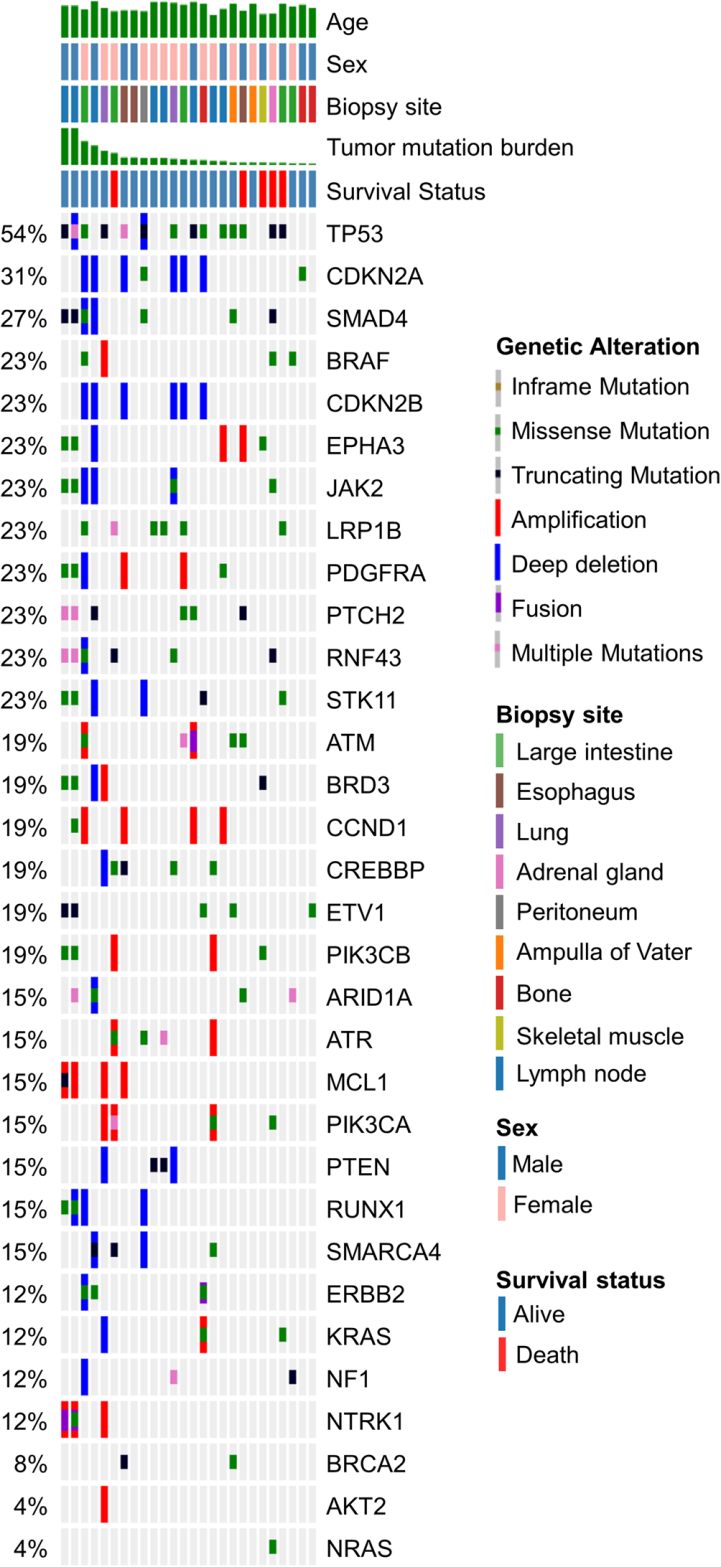
Genetic alterations in patients with CUP.

Twenty-two (10.1%) patients underwent NGS, and the most common tissues used for NGS were lymph nodes (59%), bone (18%), and lung (9%). A total of 600 alterations were identified in 268 genes (single nucleotide variation [SNV], n = 203; Insertion and Deletion [InDel], n = 68; amplification, n = 137; loss, n = 189; structural variation [SV], n = 3), and all patients showed at least one or more gene alterations. Figure 1 shows the Oncoprint of the most common genetic alterations found in the study patients. Each row and column represent each genetic alteration and patient, respectively. The most common gene alterations were TP53 (54%), CDKN2A (31%), and SMAD4 (29%). Level 1 alteration was seen in one patient who had an SV of LMNA(1q22)-NTRK1(1q23.1) fusion. One patient who had NTRK alteration initially presented with enlarged intra-abdominal lymph nodes (paraaortic, aortocaval, and small bowel mesentery area) and the results of endoscopic ultrasound-guided biopsy confirmed a moderately differentiated metastatic adenocarcinoma. Level 2 alteration was seen in three patients, which included BRAF V600E (n = 1) and BRCA (n = 2) mutations. Level 3 and 4 alterations were observed in six and five patients, respectively (Table 2). The details of NGS analysis and pathologic information of patients are provided in Supplementary Table S1.

**Table 2:** Level 2, 3, and 4 alterations in patients with CUP.

Alteration level	Alteration	N	Histology
Level 1	NTRK1-LMNA	1	Adenocarcinoma
Level 2	BRAF V600E	1	Carcinoma NOS
	BRCA2 E3167D	1	Adenocarcinoma
	BRCA2 I1859Kfs*3	1	Squamous cell carcinoma
	Total	3	
Level 3	NRAS G12D	1	Adenocarcinoma
	PIK3CA E545K	2	Squamous cell carcinoma
	ATM Q754*	1	Carcinoma NOS
	ATM K2749I	1	Carcinoma NOS
	ERBB2 S310F	1	Adenocarcinoma
	Total	6	
Level 4	BRAF G469A, NF1 R1362*	1	Adenocarcinoma
	KRAS G12A	1	Neoplasms NOS
	KRAS G12S^a^	1	Adenocarcinoma
	PTEN Q17*	2	Adenocarcinoma, squamous cell carcinoma
	Total	5	

Reference^23^.

*NOS* not otherwise specified.

^a^Also has ERBB2-PPP1R1B (level 2 alteration).

### Treatment patterns: overall and cytotoxic chemotherapy

**Figure 2:**
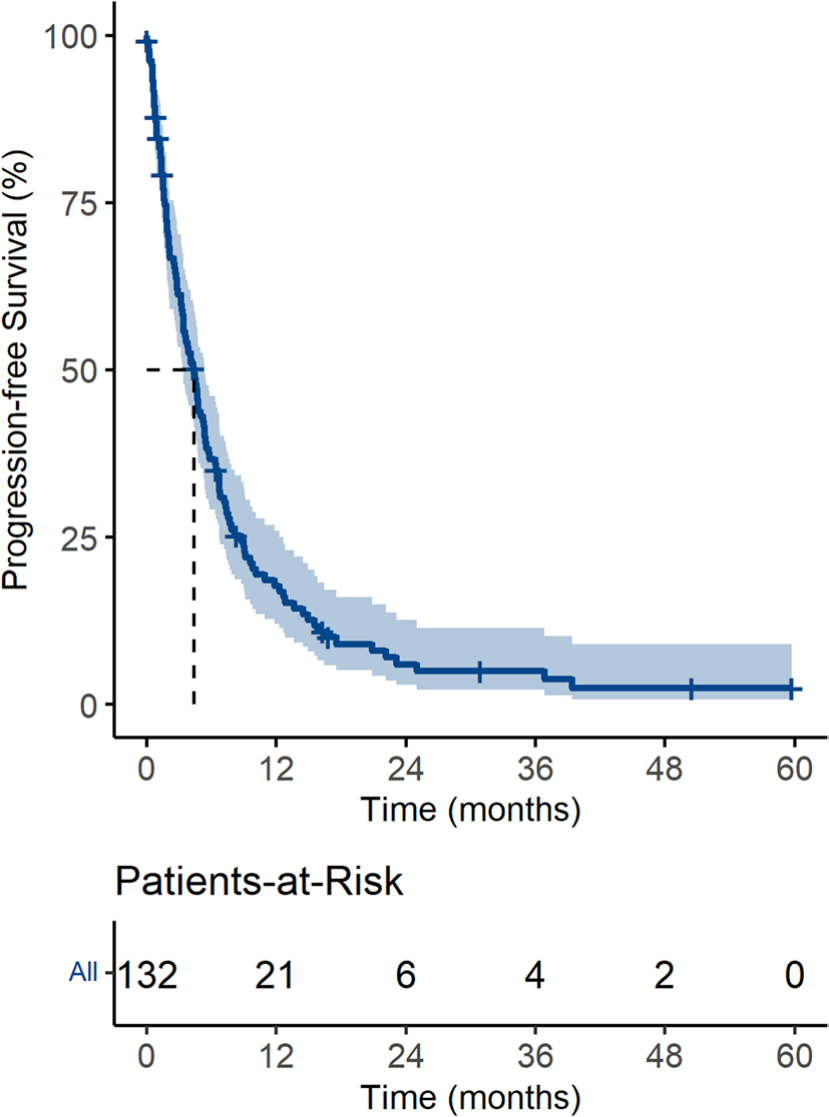
Progression-free survival in patients with CUP treated with chemotherapy.

Among the 218 study patients, 132 (60.7%) received first-line chemotherapy (Table 3), among whom 70 (53%), 28 (21%), and 6 (5%) were treated with second-, third-, and fourth-line chemotherapy, respectively. Details of the first- and second-line chemotherapy regimens are shown in Table 4. The most common regimen used as first-line chemotherapy was FP (5-fluorouracil [FU] and cisplatin, n = 74), followed by etoposide and cisplatin (EP, n = 20) and paclitaxel and carboplatin (PC, n = 13). Responses to first-line chemotherapy were as follows: complete remission (CR), n = 2; partial response (PR), n = 18; stable disease (SD), n = 28; progressive disease (PD), n = 53; and non-evaluable, n = 31; the overall response rate was 15%. PFS after first-line chemotherapy was 4.4 months (95% confidential interval [CI]) 3.4–5.3, Fig. 2). The most common reason for the discontinuation of first-line chemotherapy was disease progression (27.3%).

**Table 3:** Treatment patterns.

Treatments	Total (n = 218)
Chemotherapy	132 (60.5)
**Operation**	46 (21.1)
Diagnostic purpose	17 (36.9)
Curative resection	13 (28.3)
Palliative purpose	14 (30.4)
**Radiotherapy**	66 (30.2)
Adjuvant/definite^a^	19 (28.8)
Palliative	47 (71.2)
No treatment	58 (26.6)

Values are n (%).

^a^Includes concurrent chemo-radiotherapy (n = 10).

**Table 4:** First-line and second-line chemotherapy regimens used in patients with CUP.

Chemotherapy regimen	n (%)
**First-line (n = 132)**	
FP (5-FU, cisplatin)	74 (56.1)
EP (etoposide, cisplatin)	20 (15.2)
PC (paclitaxel, carboplatin)	13 (9.8)
Clinical trial	4 (3.0)
GP (gemcitabine, cisplatin)	3 (2.3)
VIP (cisplatin, etoposide, ifosfamide)	3 (2.3)
EC (etoposide, carboplatin)	2 (1.5)
FEP (5-FU, etoposide, cisplatin)	2 (1.5)
FOLFIRI (5-FU, leucovorin, irinotecan)	1 (0.8)
FOLFOX (5-FU, leucovorin, oxaliplatin)	1 (0.8)
TP (carboplatin, cisplatin)	1 (0.8)
Others^a^	8 (6.1)
**Second-line (n = 69)**	
PC (paclitaxel, carboplatin)	12 (17.4)
GP (gemcitabine, cisplatin)	11 (15.9)
CAV (cyclophosphamide, doxorubicin, vincristine)	9 (13.0)
FP (5-FU, cisplatin)	7 (10.1)
CYVADIC (cyclophosphamide, vincristine, doxorubin, dacarbazine)	5 (7.2)
Paclitaxel	4 (5.8)
Docetaxel	3 (4.3)
FOLFOX (5-FU, leucovorin, oxaliplatin)	3 (4.3)
CAP (cyclophosphamide, doxorubicin, cisplatin)	2 (2.9)
EP (etoposide, paclitaxel)	2 (2.9)
Pembrolizumab	1 (1.4)
Entrectinib	1 (1.4)
Others^b^	9 (13.0)

*CUP* carcinoma of unknown primary, *FU* fluorouracil.

^a^Includes casodex/lucrin, CHOP (cyclophosphamide, doxorubicin, vincristine, prednisolone), DFP (docetaxel, 5-FU, CDDP), gemcitabine, octreotide, and CVD (cyclophosphamide, vincristine, dacarbazine) (n = 1 each).

^b^Includes XELOX (oxaliplatin, capecitabine), afinitor, gemcitabine, irinotecan, IP, VIP (etoposide, ifosfamide, cisplatin), XP (capecitabine, cisplatin) + herceptin, ICE (ifosfamide, carboplatin. etoposide), and AP (doxorubicin, cisplatin) (n = 1 each).

The most common regimen used as second-line chemotherapy was PC (n = 12), followed by GP (gemcitabine and carboplatin, n = 11), CAV (cyclophosphamide, doxorubicin, and vincristine, n = 9), and FP (n = 7). The best response to second-line chemotherapy was PR (8.5%), and 42.8% of patients showed PD. The median PFS after second-line chemotherapy was 2.1 months (95% CI 1.9–4.0). The details of third-line and fourth-line chemotherapy regimens are summarized in Supplementary Table S2.

A total of 58 (26.6%) patients did not receive treatment in our center. Among them, 20 were transferred to other hospitals after diagnosis due to the patient’s choice, 11 refused treatment, 23 were unable to receive treatment due to poor PS, and 4 were lost to follow-up after the initial diagnostic work-up.

### Treatment patterns: targeted therapy

Targeted therapy was provided to two patients based on the NGS results. A patient who had NTRK fusion initially presented with abdominal pain, and CT scan showed enlarged, multiple abdominal lymph nodes in the para-aortic, aortocaval, and small-bowel mesentery areas. The pathology of the abdominal lymph node was confirmed as metastatic adenocarcinoma, and the patient was diagnosed with CUP because the primary site could not be determined through standard diagnostic evaluation. The patient was initially treated with conventional cytotoxic chemotherapy (gemcitabine-cisplatin); however, a new metastatic lung nodule was documented after 3 months (five cycles), and was thus treated with entrectinib, an FDA approved targeted therapy for solid tumor with NTRK fusion, as second-line therapy. The patient maintained a stable disease status for 9 months while taking entrectinib, but the disease had progressed as enlarged abdominal lymph nodes. As of this writing, the patient is currently participating in a clinical trial of an immune checkpoint inhibitor (spartalizumab) as third-line therapy.

The other patient who received targeted therapy had an AKT2 gain mutation and was treated with ipatasertib, an FDA-approved targeted therapy against AKT. The patient was diagnosed with CUP with involvement of the liver, lung, right ureter, and multiple lymph nodes (retroperitoneal, mediastinal, left supraclavicular) and was initially treated with conventional chemotherapy of PC and GP; however, the patient showed progression despite chemotherapy and was started on ipatasertib, but died after 2 weeks of treatment due to liver failure.

### Treatment patterns: immunotherapy

Immunotherapy was provided to three patients. One patient with inguinal area lymphadenopathy and squamous cell carcinoma was treated with PC as first-line chemotherapy. After six cycles of PC, he remained in the SD status for 6 months during the drug holiday. The disease progressed afterward and pembrolizumab was administered as second-line chemotherapy considering the microsatellite instability (MSI)-high status observed in immunohistochemistry. The PFS was approximately 6 months during pembrolizumab therapy, and FP and re-do PC were administered as third- and fourth-line chemotherapy regimens, respectively. The patient died due to septic shock and pneumonia.

Another patient treated with immunotherapy had anterior mediastinal mass, and biopsy showed poorly differentiated adenocarcinoma. Despite treatment with VIP (etoposide, ifosfamide and cisplatin), AP (doxorubicin, cisplatin), IP (irinotecan, cisplatin), and palliative radiation therapy, the disease progressed. Pembrolizumab was administered once as fourth-line chemotherapy, but the patient died within 2 weeks before the scheduled disease evaluation.

The last case is the patient who was treated with entrectinib described above in the Sect. “Treatment patterns: targeted therapy” paragraph. Currently, the patient is participating in a clinical trial of spartalizumab (anti-PD1 Ab).

### Treatment patterns: surgery

Forty-six patients underwent surgery (diagnostic surgery, n = 17; curative intent, n = 13; palliative purpose, n = 14). Surgeries for palliative purposes included the following: decompression operation due to spinal bone metastasis, bowel resection due to obstruction, internal fixation of humerus due to pathologic fracture related to bone metastasis, abdominal tumor mass excision for pain control, and brain tumor resection.

### Treatment patterns: radiotherapy

A total of 66 patients received RT. Among them, 19 received definite RT, of whom 10 underwent concurrent chemoradiotherapy (CCRT), and the rest (n = 47) received palliative RT for pain control. The diagnosis of the ten patients treated with CCRT included squamous cell carcinoma in the head and neck lesion (n = 5), squamous cell carcinoma at the mediastinum (n = 1), squamous cell carcinoma in the lymph node on the inguinal lesion (n = 1), and poorly differentiated carcinoma at the cervical lymph node region (n = 3). The median OS of the patients treated with CCRT was 51.7 months (95% CI 40.4–not reached [NR]).

### Clinical outcomes and prognostic factors

**Figure 3:**
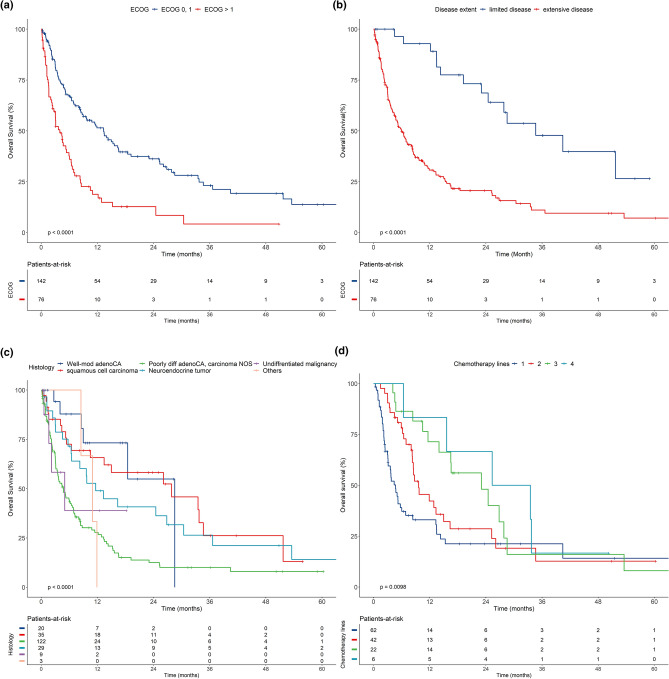
Overall survival according to (**a**) ECOG, (**b**) disease extent, (**c**) histologic groups, (**d**) number of chemotherapy lines.

The median OS of the study patients as a whole was 8.3 months (95% CI 6.2–11.4) (Supplementary Fig. S1). When divided according to the ECOG PS, those with ECOG PS 0 or 1 had a median OS duration of 13.3 months (95% CI 9.0–18.5) and those with ECOG PS greater than 1 had a median OS duration of 3.9 months (95% CI 2.7–6.0) (Fig. 3a). The OS according to disease extent is shown in Fig. 3b. The median OS duration was 34.6 months (95% CI 24.5–NR) and 6 months (95% CI 4.7–8.3) for localized disease and disseminated disease, respectively. Figure 3c shows the survival curves for patients classified by histology, and those with squamous cell carcinoma showed better outcomes than did patients with other histologic types (median OS, 27.8 months; 95% CI 13.4–NR). Patients with carcinoma NOS and poorly differentiated adenocarcinoma showed the worst survival outcomes (median OS, 4.7 months; 95% CI 3.5–6.8). However, squamous cell carcinoma was not a significant prognostic factor in subgroup analysis according to disease extent (Supplementary Fig. S2), and the median OS was 4.7 months (95% CI 3.1–8.4) for patients who only received first-line chemotherapy and 9.6 months (95% CI 8.3–16.3) for those who received second-line chemotherapy. Furthermore, the median OS was 23 months (95% CI 14.0–NR) for patients who received third-line chemotherapy and 29.4 months (95% CI 15.6–NR) for those who received fourth-line chemotherapy (Fig. 3d).

We performed univariate and multivariate Cox regression tests to identify the prognostic factors significantly related to survival outcomes in patients with CUP. In univariate analysis, ECOG PS (hazard ratio [HR], 2.47; 95% CI 1.76–3.48; P < 0.001) and localized disease (HR, 3.71; 95% CI 2.12–6.50; P < 0.001) were significantly related to better OS, whereas old age (> 60) (P = 0.45) and male sex (P = 0.96) were not significantly associated with survival outcomes. Multivariate analysis also showed that ECOG PS (HR, 2.25; 95% CI 1.59–3.17; P < 0.001) and localized disease (HR, 3.55; 95% CI 2.02–6.25; P < 0.001) were significantly related to survival outcomes (Table 5).

**Table 5:** Univariate and multivariate Cox-regression analyses of prognostic factors for overall survival in patients with CUP.

Factor	Univariate		Multivariate	
	HR (95% CI)	P value	HR (95% CI)	P value
Sex (male)^a^	0.87 (0.62–1.22)	0.45		
Age (age < 60)^a^	0.99 (0.71–1.37)	0.96		
ECOG PS (ECOG PS 0.1)^a^	2.47 (1.76–3.48)	< 0.001	2.25 (1.59–3.17)	< 0.001
Disease extent (localized disease)^a^	3.71 (2.12–6.50)	< 0.001	3.55 (2.02–6.25)	< 0.001

*CI* confidence interval, *HR* hazard ratio, *ECOG PS* Eastern Cooperative Oncology Group Performance Status.

^a^Reference.

## Discussion

In this study, we analyzed the clinical and molecular characteristics of patients with CUP and their survival outcomes in a real-world setting. Most patients initially showed metastatic disease, and the commonly involved sites were lymph nodes, liver, bone, and lung. Empirical cytotoxic chemotherapy was the most common therapeutic strategy, and surgery and radiation therapy played an auxiliary role to chemotherapy. Due to the absence of a standard chemotherapy regimen for CUP, various types of regimens were administered. Among them, platinum-based chemotherapy was the most common. Only a few patients were treated with immunotherapy and/or targeted therapy based on the NGS results. The survival outcome after standard treatment was poor.

Although the overall survival outcome of CUP was poor, subgroups of patients who had localized disease treated with CCRT demonstrated favorable outcomes (median OS, 51.7 months). This is likely because most of such patients were favorable subsets of CUP such as squamous cell carcinoma involving cervical lymph nodes and inguinal adenopathy^3^, and they showed good response to local treatment. Moreover, this may explain why patients with squamous cell carcinoma showed better survival outcomes than did patients with other histologic types.

Some retrospective studies have been carried out on the treatment patterns and outcomes of CUP. Löffler et al. analyzed the clinical characteristics, treatment patterns, and survival outcomes of 223 patients in Germany with a CUP of adenocarcinoma or un-differentiated carcinomas, and reported that the most commonly involved organ system was the lymph node, liver, bone, and lung^13^. They also found that the number of the metastatic organ systems was significantly related to survival outcomes whereas age and sex did not show such relations with survival. These results are consistent with our findings in that PS and disease extent are important factors for prognosis prediction in CUP. However, the study by Löffler et al. was different from our research in that it only included patients with adenocarcinoma or poorly differentiated carcinoma. Interestingly, Löffler et al. reported a median overall survival of 16.5 months, which is a better survival outcome than those in previous publications^3,14^ and ours. Considering that localized disease status was significantly associated with better survival outcomes in our study, such a difference in the OS results was possibly due to the differences in the proportion of patients with single organ involvement (49% vs. 15%).

Another large-scale study that included 4,562 patients using American Surveillance, Epidemiology, and End Results-Medicare (SEER-M) linked database was recently published^15^. They showed recent trends in the diagnostic work-up and treatment strategy in real-world settings and presented the patient characteristics, use of diagnostic work-up, and survival outcome. Notably, a considerable proportion of 99 (2.2%) patients received targeted therapy. The OS of all patients was poor at a median OS of 1.2 months, and only 20.3% of patients were confirmed to be alive after 6 months; such poor survival outcome may have been due to the relatively old age of patients and the low proportion of properly treated patients. In contrast, our study showed a better overall survival of 8.2 months and a higher proportion of patients received anticancer treatment. Even with recent advances in diagnostic methods and treatment strategies, the prognosis of CUP is still poor as shown in our study. One of the limitations of the SEER-M-based study was the exclusion of patients aged under 66 years.

To improve the diagnostic accuracy for CUP, new approaches are being investigated. As an example, gene expression profiling was developed to determine the primary site of the CUP, and the results revealed excellent diagnostic benefits in tumor classification with an accuracy of 85%, which is comparable to that of immunohistochemistry^16,17^. However, the clinical benefits of gene expression profiling are yet to be clearly demonstrated^10,11^, and the method is not routinely recommended for diagnostic evaluation in patients with CUP^18^.

NGS is widely used nowadays to identify actionable gene mutations in patients with CUP. In previous studies using NGS, the proportion of actionable gene mutations in CUP patients ranged from 30 to 85%^4,5,19–22^. Such a wide range of the proportion of patients with an actionable gene mutation may be due to differences in NGS assays, gene panels, and the definition of actionable mutation in each study. In our study, we used the OncoKB data for classification^23^, and 10 (43.5%) out of 23 patients showed level 1, 2, or 3 alterations.

With advances in diagnostic methods such as NGS, new treatment strategies are also being suggested. Some studies conducted NGS in CUP patients and suggested the possibility of personalized therapy based on NGS results^4,20^. In 2017, Varghese et al. reported the outcome of targeted therapy in patients with CUP based on the NGS results; of the 150 patients who underwent NGS, 45 showed clinical genomic alteration, and 10% (n = 15) received targeted therapy and showed varying treatment outcomes (time-to-treatment failure, 1–14 months)^5^. In a study conducted in South Korea, 17 among 21 patients who underwent NGS showed possible clinical genomic alterations and only one received targeted therapy^24^. More recently, a phase 2 trial of site-specific therapy based on NGS results was conducted in 97 patients with CUP; the study showed that the 1-year survival probability was 53% and the median OS was 13.7 months, thus suggesting the possible clinical applicability of tailored therapy^25^. The CUPISCO study (NCT03498521) is currently ongoing, which is a randomized trial comparing individualized targeted treatment or immunotherapy with standard platinum-based chemotherapy in patients with CUP; the results of the CUPISCO study are expected to be released within a few years.

In South Korea, NGS for patients with malignancies has been approved and reimbursed by the National Health Insurance Service since March 2017. In our study, 23 patients underwent NGS, most of whom were diagnosed after 2017. Of note, in our cohort, targeted therapy based on NGS results was provided to two patients and showed varying survival outcomes. While one patient died despite 2 weeks of treatment with ipatasertib, another patient treated with entrectinib had a better survival outcome than that of patients treated with standard empirical chemotherapy. The reason for such different clinical outcomes is likely the small number of patients who received targeted therapy, and further large-scale prospective studies are warranted to determine the role of targeted therapy.

This study has several limitations. First, the NGS panel that was used in our institution was a targeted panel sequencing for solid tumors that identifies genomic alterations in approximately 300 cancer-related genes. As such, some gene alterations that were not included in our panel would not have been identified. This may be a reason for the low proportion of patients who were treated with targeted therapy, and it may have affected the clinical outcomes in our cohort.

Also, our NGS results could not suggest proper treatments for most of the patients in our cohort. We believe that this reflects the difficulties in real-world practice that optimal targetable gene alterations are hard to identify, even with NGS assays, due to the presence of multiple concurrent gene mutations in CUP patients and the limitation of the NGS assay described above. Furthermore, even if we found actionable gene mutation using NGS assay, the targeted agents for most of the actionable mutations are not readily available for clinical use, and this also may be related the unsatisfactory outcomes in patients with CUP. In order to improve the clinical outcomes of CUP patients, further investigations are needed to develop the proper method for evaluating targetable genetic alterations and to develop optimal targeted therapies.

Second, as this study was conducted at a single center, there may have been selection bias and the results may have limited generalizability. The number of study patients was small, but it is a relatively large-scale study considering the rare prevalence of the CUP, and there has not been a study of this scale in Asian patients with CUP. Moreover, despite its retrospective nature, this study is meaningful because a prospective study cannot be easily conducted due to the nature of the disease and our results reflect the current trends and outcome in a real-world setting. Another limitation is that we could not systematically evaluate the adverse effects in each patient due to the heterogeneity of the chemotherapy regimen.

## Materials and methods

Patients who were diagnosed with CUP and registered in our institution’s cancer registry between January 2009 and December 2019 were identified and included in the study. CUP was defined according to the initial ICD code of “unknown primary site” (ICD-0-3 code 80.9). Among them, after a comprehensive chart review, we excluded patients whose primary site was later identified through further imaging studies and/or histologic diagnosis with additional immunohistochemical staining. We reviewed the medical records of all included patients to collect data on demographic and clinical characteristics including age, sex, ECOG-PS, histopathological diagnosis, number and location of metastases, disease extent, treatment strategy, chemotherapy regimen, response to chemotherapy, and survival outcome. Disease extent was classified into localized disease (i.e., single lymph node region or single site) and disseminated disease. All non-localized cases were classified as disseminated diseases.

### NGS and genomic analysis

Genomic DNA was extracted from previously archived tumor tissues. Targeted sequencing was carried out using the MiSeq platform (Illumina, San Diego, CA, USA) with an in-house panel designed at Asan Medical Center (OncoPanel AMC, versions 3 and 4) using the SureDesign (Agilent Technologies, Santa Clara, CA, USA) with the GRCh37 reference version. The Oncopanel AMC version 3 and 4 (OP AMC v3 and v4) identifies genomic alterations in 383 and 323 cancer-related genes, respectively; specifically, the OP AMC v3 examines 199 genes for SNV/INDEL, and copy number variation (CNV), 8 genes for rearrangement, and 184 hotspots, and the OP AMC v4 examines 225 genes for SNV/INDEL and CNV, 6 genes for rearrangement, and 99 hotspots. The included gene lists are provided in Supplementary Table S3 for OP AMC v3, and Supplementary Table S4 for OP AMC v4. The sequence mapping steps for OP AMC v3 and v4 were carried out according to the methods described in a previous report^26^. VarDict was used to conduct somatic variant calling for single nucleotide variants and short indels^27^. CNVkit was used to perform Copy Number analyses^28^. Using common germline variants database (dbSNP build 141 [found in 1% > of samples]^29^, Exome Aggregation Consortium release 0.3.1^30^, and Korean Reference Genome database^31^), common and germline variants candidates from somatic variants were extracted. We identified patients who underwent NGS and analyzed their NGS results. The clinical actionability of specific molecular alterations was assessed using OncoKB, a precision oncology knowledge database (http://oncokb.org)^23^. According to the OncoKB database, actionable gene alterations were classified from levels 1 to 4: level 1 alterations are defined as Food and Drug Administration (FDA)-recognized biomarkers for FDA-approved drugs in a specific cancer, level 2 alterations are defined as standard biomarker recommended by the standard guidelines for an FDA-approved drug, level 3 alterations are defined as biomarker supported by clinical evidence as being predicted of response to a certain drug, and level 4 alterations are defined biomarkers supported by biological and preclinical evidence^23^.

### Statistical analysis

OS was calculated from the date of pathological diagnosis to the date of death due to any cause. Progression-free survival (PFS) was calculated from the date of chemotherapy initiation to the date of documented progression or death. If a patient was alive on the date of the last outpatient visit and lost to follow-up, we censored the patient at the date of the last outpatient visit date. The Kaplan–Meier method was used to calculate the OS and PFS, and the log-rank method was used to compare the OS and PFS between subgroups. Multivariate Cox regression analysis was performed to determine the prognostic value of variables. P values < 0.05 were considered statistically significant. All statistical analyses were conducted using R statistical software, version 3.6.3 (R Foundation for Statistical Computing, Vienna, Austria)^32^.

### Ethical approval

This retrospective study was performed in accordance with the Declaration of Helsinki, and the protocol was reviewed and approved by the Institutional Review Board (IRB) of Asan Medical Center (approval number: 2016-0491, date of approval: May 10, 2016). Written informed consent was obtained from study participants who underwent NGS, and the requirement of obtaining informed consent from patients who did not conduct NGS was waived by IRBs because of the retrospective nature of this study.

## Supplementary Information

Supplementary Information.
