# Supplementary material for: Real-world data analysis of patients with cancer of unknown primary
Source: Sci Rep. 2021 Nov 29;11:23074. doi: 10.1038/s41598-021-02543-1 (PMC8630084; doi:10.1038/s41598-021-02543-1)

Supplementary Table Legends

Table S1. Pathologic information and gene mutation of patients who underwent NGS.

Table S2. Third-line and fourth-line chemotherapy regimens used in patients with CUP.

Table S3. DNA gene list of OncoPanel AMC version 3.

Table S4. DNA gene list of OncoPanel AMC version 4.

Supplementary Figure Legends

Figure S1. Overall survival in patients with CUP

Figure S2. Overall survival according to histology in (a) patients with extensive disease and (b) patients with localized disease

Table S4. Pathologic information and gene mutation of patients who underwent NGS.

ID	Anatomical location	Histology	Sex	Age	CK7	CK20	Other IHC stain	Pathological hypothesis	Gene mutation founded by NGS
**1**	LN (Lt paraaortic, aortocaval, Small bowel, mesentery area)	AdenoCA	M	66	NEG	NEG	TTF-1$^-$ CDX2$^+$	Pancreatobiliary	LMNA-NTRK1 fusion<br>PALB2 T993M<br>TP53 V73Wfs*50<br>RNF43 G659Vfs*41, L311Sfs*108<br>POLE R266*<br>SMAD4 K110Nfs*12
**2**	Bone (skull, right 7th, left 8th ribs, T8, T10, T12, L4, right ischium, left pelvic bone)	NEC	M	49	ND	ND	CD138$^-$<br>CD31$^-$ CD34$^-$<br>CD56$^+$<br>CD, AE1/AE3$^-$<br>ERG$^-$ PAX-8$^+$<br>SMA$^-$ S100$^+$<br>Synaptophysin$^+$<br>CD99$^-$ HHV-8$^-$	Malignant paraganglioma	POLE T41M<br>RB1 K122E<br>AXIN1S57L<br>PIK3CB R321Q
**3**	LN (left cervical, left supraclavicular, retroperitoneum, both iliac)	SqCC	F	73	ND	ND	p40$^+$	ND	PTEN Q17*<br>IGF1R R252H
**4**	LN (Lt. supraclavicular, mediastinal, retroperitoneal, pelvic) Bone (C6, T1)	SqCC	F	45	ND	ND	p40$^+$	Cervix origin	PIK3CA E545K<br>AKT3 L313P<br>BAP1 S63C<br>NOTCH4 G1623R<br>CREBBP L74V<br>SMARCA4 L1073Q
**5**	Peritoneal carcinomatosis	AdenoCA	F	70	POS	POS	TTF-1$^-$ CDX-2$^+$	Gastrointestinal	SMAD4 P356L<br>TP53 V73M<br>BRCA2 E3167D
**6**	Left adrenal gland, LN (retroperitoneum, left supraclavicular)	AdenoCA	F	50	POS	POS	CDX-2$^+$	Gastrointestinal	NRAS G12D<br>BRAF G466V<br>TP53 E68*,<br>RNF43 R145*

									SMAD4 Q442*
**7**	Lung, LN (both lung), right kidney, proximal ureter	Carcinoma NOS	F	59	POS	ND	TTF-1^{-} GATA-3^{-} WT-1^{-} PAX-8^{-} p63$^{+/-}$ S-100P^{-} ER^{-} p53$^{+/-}$	High grade urothelial carcinoma	Loss of PTEN<br>Gain of AKT2
**8**	Bone (Skull base, C-T-L-S spine, both humeri, sternum, both clavicles, both scapulae, both side ribs, both pelvic bones), lung, LN (Rt. cervical)	Carcinoma NOS	M	61	NEG	ND	TTF-1^{-} AMACR^{+} PAP^{-} PAS^{-}	Prostate	RICTOR amplification
**9**	LN (left supraclavicular, bilateral retrocrural, left gastric, common hepatic, hepatoduodenal ligament, mesenteric, retroperitoneal areas)	AdenoCA	M	59	POS	POS	TTF-1^{-} CDX-2^{-}	Lung or colon	TP53 R175G<br>amplification of 7q21.12-21.2 (CDK6, CASD1)<br>amplification of 11q13.2-13.4 (CCND1, FAM86C)
**10**	Abdominal wall mass	AdenoCA	F	52	POS	POS	SMAD4^{+} TTF-1$^{–}$ ER$^{–}$ PR$^{–}$ PAX8$^{–}$	Pancreatobiliary or stomach origin	SMAD4 R361C<br>TP53 X126_splice
**11**	Bone (left pelvic), Lung	AdenoCA	F	70	POS	NEG	CDX-2^{+}<br>P53 3+<br>PAX-8-<br>SMAD-4;intact<br>WT-1^{-}	Stomach or appendix or biliary or pelvic peritoneal	KRAS G12S<br>TP53 C238Y<br>CNV loss of CDNK2A/2B<br>ERBB2-PPP1R1B
**12**	LN (para-aortic, aortocaval, Lt. common iliac)	AdenoCA	F	57	ND	ND	ER^{-} PR^{-} Chromogranin^{-} Synaptophysin^{-}	Gastrointestinal	BRAF V600E<br>TP53 V173M<br>MYC amplification
**13**	LN (inguinal)	SqCC	F	54	ND	ND	p40^{+}	ND	PIK3CA E545K<br>FBXW7 R465C

									XRCC2 R64*<br>ARID2 N1778Ifs*13<br>RNF43 G659Vfs*41<br>SMARCA4 X473_splice<br>ACVR2A
**14**	LN (gastrohepatic ligament, porta hepatic, hepatoduodenal ligament, portocaval space, retroperitoneum)	AdenoCA	M	75	POS	NEG	TTF-1^{-}<br>GATA3^{-}<br>p63^{-}<br>CDX-2^{-}<br>PSA^{-}<br>S100^{+}	Lung or Thyroid or salivary gland or gastrointestinal tract or hepatobiliary	ERBB2 S310F<br>SMARCA4 E1023*
**15**	Bone (whole body bone), LN (right lower paratracheal)	AdenoCA	F	61	POS	ND	TTF-1^{-} ALK^{-}<br>GATA-3^{-}<br>ER^{-} PR^{-}<br>c-erb B2^{-}	Lung (ROS1 translocation)	BRAF G649A<br>ARID1A W1686*/D1850Gfs*4<br>CTNNB1 S45F<br>NF1 R1362*<br>FLT4 A1158V
**16**	Scalp mass, Lung, intraabdominal mass near the ureter	AdenoCA	F	72	POS	POS	p63^{-}<br>PAX-8^{-}<br>TTF-1^{-}	Pulmonary enteric type carcinoma or colorectal	NF1 H1943Lfs*19<br>TP53 R175H<br>ARID2 K706* T1540Nfs*4<br>Loss of PTEN<br>Loss of CDKNA2A/2B
**17**	Neck mass, LN (retropharyngeal, right intraparotid, cervical, supraclavicular, paratracheal)	SqCC	M	55	ND	ND	p16^{-}<br>EBV(ISH)$^{-}$<br>HPV^{-}<br>TTF-1^{-}	Head and neck	NOTCH1 E18278*<br>BRCA2 I1859Kfs*3<br>CREBBP Q955*<br>TP53 G266*, R213Kfs*7<br>CCND1 amplification<br>Loss of CDKN2A/2B
**18**	Bone (right occipital base, C7, T4, right scapula, right 2nd, 3rd rib)	Carcinoma NOS	M	68	POS	NEG	PSA^{-} TTF-1^{-}<br>Synaptophysin^{-}<br>CD138^{-}<br>P40^{-} CD56^{-}<br>CK, AE1/AE3^{+}<br>GATA-3^{+}	Head and Neck or Bladder	CDKN2A H66R

							HEP PAR-1$^-$<br>PAX-8$^-$		
**19**	LN (Paraaortic, aortocaval, retroperitoneum, mesentery, both iliac area)	Carcinoma NOS	M	73	POS	NEG	Chromogranin$^-$<br>CD45$^-$<br>p40$^-$ p63$^-$<br>PAP$^+$ PSA$^-$<br>CD56$^-$ SALL-4$^-$	Prostate	TP53 X307_splice<br>ATM K2749I<br>CCND1 amplification
**20**	LN (portocaval area)	Carcinoma NOS	F	68	POS	POS	AFP$^+$	Stomach or<br>liver or pancreatobiliary	ATM Q754*<br>ATM L442_Q446del<br>amplifications of 4q12 (PDGFRA, KIT, KDR)<br>Amplification of 12q13.3-15 (ERBB3, MYO1A, CDK4, MDM2)<br>Loss of CDKN2A/B
**21**	Liver, LN (cervical, retroperitoneum, aortocaval, both common and external iliac area, subcarinal, interlobar, paraesophageal) Bone (T10)	Carcinoma NOS	M	56	POS	NEG	CD30$^-$<br>CK, AE1/AE3$^+$<br>EBV(ISH)$^-$<br>HEP PAR-1$^-$<br>CD45$^-$ CD20$^-$<br>CD163$^-$ CD3$^-$<br>CD68$^-$ HMB45$^-$<br>S100$^{+/-}$<br>CK MNF116$^+$<br>EMA$^-$	ND	TP53 C242R<br>TSC N762S<br>EGFR amplification
**22**	LN (hepatoduodenal, portocaval, aortocaval, Lt. interlobar)	Carcinoma NOS	M	72	ND	ND	CK, AE1/AE3$^-$<br>S100$^-$<br>CD45$^-$ CD34$^-$<br>CD99$^+$ CD30$^-$<br>Myogenin$^-$<br>SOX-10$^-$<br>CD31$^-$ ERG$^-$<br>EMA$^{+/-}$	ND	KRAS G12A<br>TP53 R213*

FLI-1+<br>Inhibin$^-$ CD163$^-$<br>CD68$^-$ EBV(ISH)-<br>CD21$^-$ CD23$^-$<br>Mucicarmine$^-$

Abbreviations: ND, not defined; Pos, positive; Neg, negative; IHC, immunohistochemistry ; LN, lymph node; NOS, not otherwise specified; adenoCA, adenocarcinoma; SqCC, squamous cell carcinoma; NEC, neuroendocrine carcinoma

Table S2. Third-line and fourth-line chemotherapy regimens used in patients with CUP

Chemotherapy regimen	n (%)
**Third-line chemotherapy regimen (n = 28)**	
Clinical trial	5 (17.9)
FP (5-FU, cisplatin)	4 (14.3)
CAV (cyclophosphamide, doxorubicin, vincristine)	1 (3.6)
VIP (etoposide, ifosfamide, and cisplatin)	1 (3.6)
GP (gemcitabine, carboplatin)	1 (3.6)
CAP (cyclophosphamide, adriamycin, cisplatin)	1 (3.6)
CYVADIC (cyclophosphamide, vincristine, doxorubin, dacarbazine)	1 (3.6)
Docetaxel	1 (3.6)
IP (irinotecan, cisplatin)	3 (10.7)
Others*	10 (35.7)
**Fourth-line chemotherapy regimen (n = 6)**	
Gemcitabine	1 (16.6)
Ipatasertib	1 (16.6)
VIP (etoposide, ifosfamide, cisplatin)	1 (16.6)
CAV (cyclophosphamide, adriamycin, cisplatin)	1 (16.6)
PC (paclitaxel, cisplatin)	1 (16.6)
Pembrolizumab	1 (16.6)

*includes EMF, MVAC, NP, ifosfamide, FOLFIRI, AP, IT-MTX, bevacizumab/doxorubicin, AIM, and MF (n = 1 each)

Table S3. DNA gene list of OncoPanel AMC version 3

DNA gene list: Entire exonic sequence for the detection of the base substitution, insertion/deletions, and copy number alterations					
ABL1	ABL2	AKT1	AKT2	AKT3	ALK
APC	AR	ARAF	ARID1A	ARID1B	ARID2
ASXL1	ATM	ATR	ATRX	AURKA	AURKB
AURKC	AXIN1	AXL	BAP1	BARD1	BCL2
BRAF	BRCA1	BRCA2	BRD2	BRD3	BRD4
BRIP1	CBFB	CCND1	CCND2	CCND3	CCNE1
CD274	CDH1	CDK12	CDK4	CDK6	CDKN1A
CDKN1B	CDKN2A	CDKN2B	CDKN2C	CEBPA	CHEK2
CREBBP	CRKL	CSF1R	CTNNB1	DDR1	DDR2
DNMT3A	DOT1L	DPYD	EGFR	EPHA3	EPHB4
ERBB2	ERBB3	ERBB4	ERCC2	ERG	ERRFI1
ESR1	ETV1	ETV4	ETV5	ETV6	EWSR1
EZH2	FAM175A	FBXW7	FGFR1	FGFR2	FGFR3
FGFR4	FLCN	FLT1	FLT3	FLT4	FOXL2
GATA2	GEN1	GNA11	GNAQ	GNAS	HDAC9
HGF	HNF1A	HRAS	IDH1	IDH2	IGF1R
IGF2	JAK1	JAK2	JAK3	KDR	KIT
KMT2A	KRAS	LRP1B	MAP2K1	MAP2K2	MAP2K4
MAP3K1	MAP3K4	MAPK1	MAPK3	MAPK8	MCL1
MDM2	MDM4	MED12	MEN1	MET	MITF
MLH1	MPL	MRE11A	MSH2	MSH6	MTOR
MYC	MYCN	MYD88	NBN	NF1	NF2
NFKBIA	NKX2-1	NOTCH1	NOTCH2	NOTCH3	NOTCH4
NPM1	NRAS	NTRK1	NTRK2	NTRK3	NUTM1
PALB2	PBRM1	PDGFB	PDGFRA	PDGFRB	PIK3CA
PIK3CB	PIK3CD	PIK3R1	PIK3R2	PMS2	POLE
PPARG	PTCH1	PTCH2	PTEN	PTPN11	RAB35
RAD50	RAD51	RAD51C	RAD51D	RAF1	RARA
RB1	RET	RHEB	RICTOR	RNF43	ROS1
RSPO1	RSPO2	RUNX1	SMAD2	SMAD4	SMARCA4
SMARCB1	SMO	SPOP	SRC	STK11	SYK
TERT	TET2	TMPRSS2	TOP1	TOP2A	TP53
TSC1	TSC2	VHL	WT1	XPO1	XRCC2
ZNRF3					
DNA gene list: For the detection of select rearrangements					

ABL1	ALK	EGFR	EWSR1	NTRK1	RET
ROS1	TMPRSS2				

DNA gene list: Hotspots for the detection of base substitution and insertions/deletions

A1BG	ABCC5	ACVR1	ACVR2A	ADAMTS18	ADNP
AKAP7	ALX4	ANKRD20A3	ANTXR2	AP1S1	AP3S1
ARV1	ASH1L	ATP1A1	BAX	BCL7C	BLM
BTK	CALR	CAMSAP1L1	CASD1	CBL	CBWD3
CBX4	CBX5	CCDC73	CD3G	CD79B	CDH26
CEBPZ	CENPV	CEP290	CHERP	CISD2	CKAP2
CLEC18C	CLOCK	COBLL1	CPEB2	CRIPAK	CTGF
DDX11	DHX9	DLC1	DNAH12	DOCK3	DPAGT1
DYNC1I2	EBPL	EPPK1	FABP2	FAM115C	FAM153A
FAM18A	FAM193A	FAM75A6	FAM86C1	FAT1	FBXL3
FGFBP1	FMN2	FOXD4L6	FRG2B	FXR1	GOLGA8B
GOLGA8R	GRIN3B	GTPBP2	HIAT1	HIF1A	HS6ST1
HSPD1	IFITM1	IFITM3	IL10RB	IMPA1	INO80E
IRS1	KCTD16	KIAA1919	KLRC3	KNSTRN	KRT32
LIPT1	LMBRD1	MADCAM1	MAX	MBD3L3	MBD3L4
MTHFR	MUC12	MUC2	MVK	MYBL1	MYO1A
NBPF16	NBPF9	NDUFA6	NFE2L2	NGLY1	NIPA2
NNAT	NOMO1	NOS3	NOTCH2NL	NPEPPS	NUDT7
OR1D5	OR2T29	OR2T35	OR4M2	OR5B17	OR6B2
PABPC1	PCBP1	PCDHB16	PCDHB4	PCMTD1	PDXDC1
PMS2P11	POM121L3	PON2	PPP2R1A	PRAMEF11	PRAMEF13
PRAMEF19	PREX2	PRIM2	PSPC1	PTH2	PTPN11
PTPN3	RAC1	RASA4	RBBP8	RFX5	RGS12
RHOA	RNF222	RPN1	RRN3	RUFY2	SAA2
SAFB2	SCAF4	SEC63	SELRC1	SF3B1	SF3B5
SLC23A2	SPRR3	SSTR4	STAMBPL1	STAT3	STAT6
STAU2	SULT1A1	SULT6B1	SYCP1	SYNJ2	TAS2R19
TAS2R31	TBC1D3E	TCF7L2	TEAD2	TIMM23	TMEM14B
TMEM60	TMPRSS13	TNPO1	TPSAB1	TPSD1	U2AF1
UPK3BL	WDR55	WDR87	XAB2	ZBTB7C	ZFP37
ZNF141	ZNF198	ZNF518A	ZNF563		

Table S4. DNA gene list of OncoPanel AMC version 4.

DNA gene list: Entire exonic sequence for the detection of the base substitution, insertion/deletions, and copy number alterations					
ABL1	ABL2	AKT1	AKT2	ALK	APC
AR	ARAF	ARID1A	ARID1B	ARID2	ASXL1
ATM	ATR	ATRX	AURKA	AURKB	AURKC
AXIN1	AXL	BAP1	BARD1	BRAF	BRCA1
BRCA2	BRD2	BRD3	BRD4	BRIP1	CBFB
CCND1	CCND2	CCND3	CCNE1	CD274	CDH1
CDK12	CDK4	CDK6	CDKN1A	CDKN1B	CDKN2A
CDKN2B	CDKN2C	CEBPA	CHEK2	CREBBP	CSF1R
CTNNB1	DDR1	DDR2	DDX3X	DNMT3A	DOT1L
DPYD	EGFR	EPHA3	EPHB4	ERBB2	ERBB3
ERBB4	ERCC2	ERCC4	ERG	ERRFI1	ESR1
ETV1	ETV4	ETV5	ETV6	EWSR1	EZH2
FAM175A	FANCA	FANCB	FANCC	FANCD2	FANCE
FANCF	FANCG	FANCI	FANCL	FANCM	FBXW7
FGF19	FGF4	FGFR1	FGFR2	FGFR3	FGFR4
FLCN	FLT1	FLT3	FLT4	FOXL2	FUBP1
GATA2	GEN1	GNA11	GANQ	GNAS	H3F3A
HDAC9	HGF	HLA-A	HLA-B	HLA-C	HAL-DRB1
HNF1A	HRAS	IDH1	IDH2	IGF1R	IGF2
JAK1	JAK2	JAK3	KDR	KIT	KMT2A
KRAS	LRP1B	LTK	MAP2K1	MAP2K2	MAP2K4
MAP3K1	MAP3K4	MAPK1	MAPK3	MAPK8	MCL1
MDM2	MDM4	MED12	MEN1	MET	MITF
MLH1	MPL	MRE11A	MSH2	MSH6	MTAP
MTOR	MYC	MYCN	NBN	NF1	NF2
NFKB1A	NKX2-1	NOTCH1	NOTCH2	NOTCH3	NOTCH4
NRAS	NTRK1	NTRK2	NTRK3	NUMT1	PALB2
PARP1	PBRM1	PDGFB	PDGFRA	PDGFRB	PIK3A
PIK3CB	PIK3CD	PIK3R1	PIK3R2	PMS2	POLE
PPARG	PTCH1	PTCH2	PTEN	PTPN11	RAD50
RAD51	RAD51C	RAD51D	RAF1	RARA	RB1
RET	RICTOR	RNF43	ROS2	RSPO1	RSPO2
RUNX1	SDHA	SDHB	SDHC	SETD2	SLX4
SMAD2	SMAD4	SMARCA4	SMARCB1	SMO	SOX2
SOX9	SPOP	SRC	STK11	SYK	TERT

TET2	TMPR22S	TOP1	TOP2A	TP53	TSC1
TSC2	UBE2T	VHL	WT1	XPO1	XRCC2
ZNRF3					
DNA gene list: For the detection of select rearrangements					
ALK	EGFR	NTRK1	RET	ROS1	BRAF
DNA gene list: Hotspots for the detection of base substitution and insertions/deletions					
A1BG	ABCC5	ACVR2A	ADAMTS18	ADNP	AKAP7
AP1S1	ARV1A	ASH1L	BAX	BTK	CASD1
CBL	CBX4	CCDC73	CD3G	CDH26	CEBPZ
CENPV	CKAP2	CLOCK	COBLL1	CPEB2	CRIPAK
DLC1	DNAH12	DOCK3	DPAGT1	DYNC1I2	EBPL
EPPK1	FBXL3	FGFBP1	FMN2	FRG2B	FXR1
GRIN3B	GTPBP2	HIAT1	IFITM1	IFITM3	IMPA1
INO80E	IRS1	KCTD16	KIAA1919	KLF4	KNSTRN
KRT32	LIPT1	MADCAM1	MAX	MVK	MYO1A
NFE2L2	NIPA2	NNAT	NOS3	NOTCH2NL	NUDT7
OR4M2	PABPC1	PCBP1	PCDHB16	PCMTD1	PPP2R1A
PREX2	PRIM2	PTPN11	RAC1	RASA4	RBBP8
RGS12	RHOA	RUFY2	SEC63	SF3B1	SLC23A2
SPRR3	SSTR4	STAMBPL1	STAT3	STAU2	SULT6B1
SYNJ2	TAS2R19	TAS2R31	TCF7L2	TEAD2	TMEM60
TMPRSS13	TPSD1	U2AF1	WDR55	WDR87	ZFP37
ZNF141	ZNF563				

Figure S1. Overall survival in patients with CUP

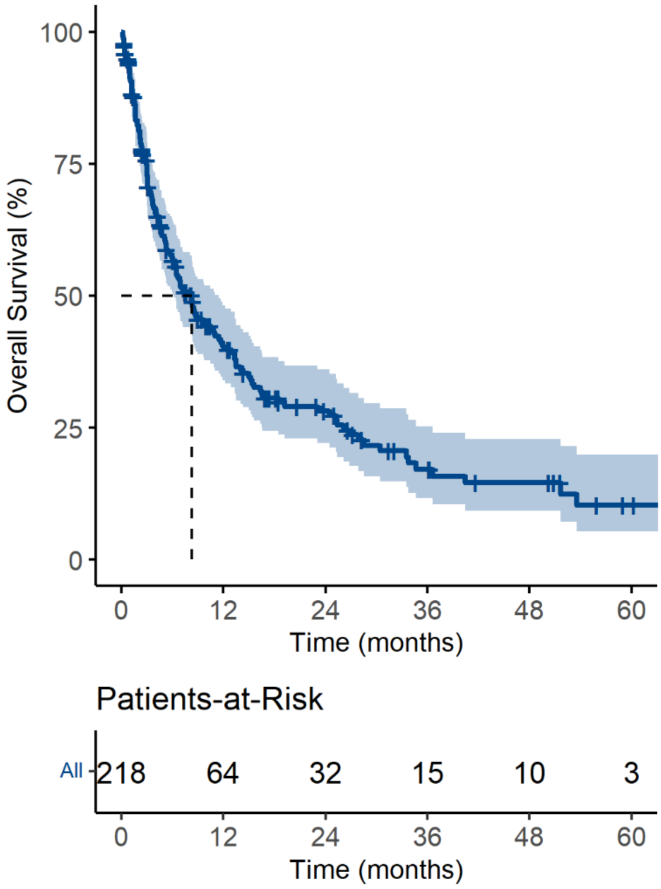

Figure S2. Overall survival according to histology in (a) patients with extensive disease and (b) patients with localized disease

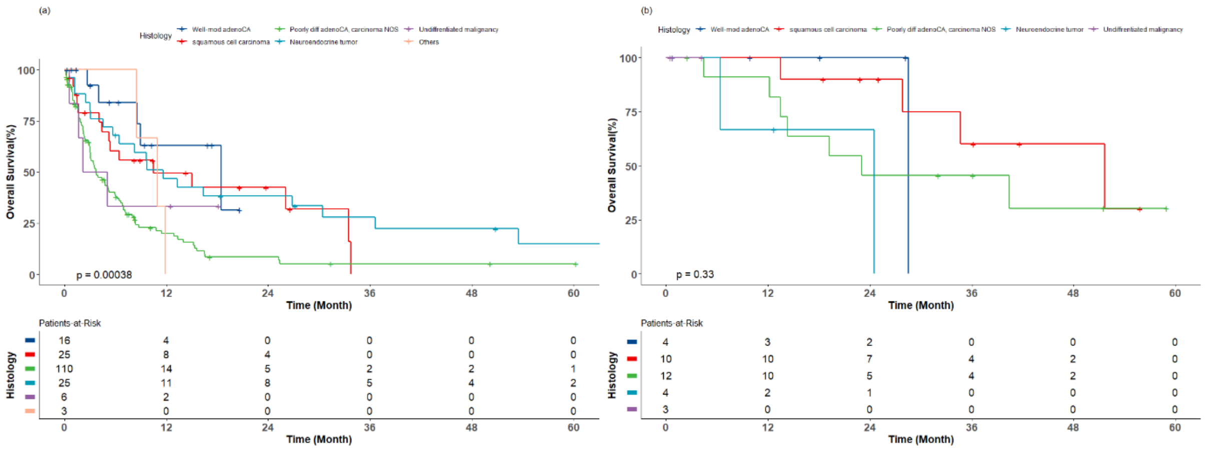

Supplement: Supplementary file 1 — Supplementary Information. [file 41598_2021_2543_MOESM1_ESM.pdf]
